# Chloroform, an Innocuous Anæsthetic

**Published:** 1854-07

**Authors:** John C. Bennett


### *Chloroform, an Innocuous Anæsthetic.*

BY JOHN C. BENNETT, M. D.

We extract the following from a paper read before the Medico-Chirurgical College, Phila., by J. C. Bennett, M. D., and published by order of the College. Dr. Bennett located at Fort Desmoines, in this State, but is now on a visit to Great Falls, N. H.

After giving a history of Chloroform and referring to the difference of opinion in regard to the comparison between ether and chloroform, he says:

"This opinion would be entitled to greater respect if the composition of chloric ether were unknown. Accurate analysis, however, shows that "concentrated chloric ether contains 33½ per cent. pure chloroform, the remainder being nearly alsolute alcohol (containing but about 4 or 5 per cent. of water.)" It is difficult, therefore, to understand in what its superiority over pure chloroform consists.—The fact is, that chloric ether is less volatile than chloroform, and therefore it produces its original effects more tardily, and its subsequent influence on the system is prolonged. The alcohol contained in it tends to the induration of the surface of the delicate membrane of the lungs, with which it comes in contact in inhalation; and on this grave account, if on no other, its use is objectional, where chloroform is admissible. The secondary effects of sulphuric ether are also to be deprecated. At all events, on the theory of the most decided enemies of chloroform, its use has not produced worse consequences than have ensued from the exhibition of calomel, antimony, and opium; and yet these, by the same parties, are constantly lauded as the chief reliance of the healing art.

Francis Gurney Smith, M. D., editor of the Medical Examiner, of Philadelphia, in a review of the transactions of the American Medical Association, vol. ii., says:

"The superiority of chloroform over ether is sustained by the testimony of a number of eminent American surgeons of large experience, whose views were solicited, and altogether form a strong array in favor of this powerful agent."

The truth is, that pure chloroform is safer than any other anæsthetic, and is less dangerous than any other potent article used in medicine. It is the recorded opinion of Dr. Warren, of Boston, that chloroform "has advantages over the other ethers. It seems to steal away the senses in a more quiet and insidious manner. There is less pulmonary irritation. The time necessary with chloroform for the production of these effects is less than with sulphuric ether. The degree of insensibility is, I think, greater from chloroform; as we do not often notice the unconscious shrinking which seems to indicate pain. The duration of this state is also more considerable. The restoration of nervous action is more sudden and perfect. The more sudden action of chloroform, and the more perfect termination of this action, is to be attributed probably to its greater volatility; for, although a more dense liquid than sulphuric ether, its emanations diffuse themselves more rapidly, act of course more speedily, and are more suddenly absorbed into the circulation. A great advantage of chloroform is found in the comparatively small quantity required for etherization; twenty or thirty drops, judiciously applied, are often sufficient. This fact has contributed much to its sudden and entensive use in this country. An additional recommendation is the absence of inflammability."

The extensive experience of the writer in the use of this article, entitles his opinion to much credit and respect. We entirely agree with him in the opinion that if an anæsthetic be indicated, that chloroform is to be preferred. There is no doubt but that if pure chloroform be used there would be fewer unhappy results. The article is too long to copy entire in this Journal, we have therefore selected from it some judicious and valuable hints in relation to its adulteration and the consequences of using an impure article. EDS.

